# Supplementary material for: Knowledge and attitudes about conduct disorder of professionals working with young people: The influence of occupation and direct and indirect experience
Source: PLoS One. 2023 Sep 28;18(9):e0292271. doi: 10.1371/journal.pone.0292271 (PMC10538712; doi:10.1371/journal.pone.0292271)
Supplement: S1 Appendix — The items that correspond to each of the subscales (characteristics, treatments, and causes) can be seen. Also, it states which items are true or false. Finally, Table 7 in S1 Appendix demonstrates the items and their corresponding citations, followed by a list of references. (DOCX) [file pone.0292271.s001.docx]

Appendix

List of items on objective knowledge of conduct disorder scale, corresponding citations, and scoring instructions

Subscales:

- Characteristics items: 3, 7, 10, 15, 17, 19, 21, 25, 26, 30, 33
- Treatments items: 1, 2, 5, 8, 9, 16, 18, 20, 23, 27, 29
- Causes items: 4, 6, 11, 12, 13, 14, 22, 24, 28, 31, 32

Correct answers:

- Correct answer is *true*: 1, 3, 5, 7, 9, 10, 12, 15, 16, 17, 19, 21, 26, 33
- Correct answer is *false*: 2, 4, 6, 8, 11, 13, 14, 18, 20, 22, 23, 24, 25, 27, 28, 29, 30, 31, 32

**Table 7. Items and their corresponding citations.**

| Item | Source |
| --- | --- |
| 1. Side effects for pharmacological treatments of conduct disorder include weight gain | [1,2] |
| 1. Children with callous-unemotional traits respond better to typical interventions compared to those without callous-unemotional traits | [3–5] |
| 1. Language impairments are common amongst children with conduct disorder | [6,7] |
| 1. Maternal smoking during pregnancy has no effect on the risk of conduct disorder in their offspring | [8,9] |
| Item | Source |
| 1. It is recommended that psychosocial interventions should be used as the standard treatment for conduct disorder | [10] |
| 1. Only biological factors cause conduct disorder | [11] |
| 1. It is possible for an adult to be diagnosed with conduct disorder | [12,13] |
| 1. Harsh boot camps are successful for reducing offending in young people with conduct problems | [14,15] |
| 1. Antipsychotic drugs can be used to manage symptoms of conduct disorder | [1,2,16] |
| 1. Current wisdom suggests two subgroups of conduct disorder: with limited prosocial emotions and without limited prosocial emotions | [12,17] |
| 1. Lower familial socioeconomic status relates to lower levels of conduct problems | [18–20] |
| 1. Heritability estimates demonstrate genetic factors largely contribute to conduct disorder | [21–23] |
| 1. Children are less likely to have conduct problems if their mother has depression | [24–26] |
| 1. Research shows no relationship between malnutrition at a young age and conduct disorder | [27,28] |
| 1. Children with conduct disorder are at a higher risk of engaging in criminal acts | [29–31] |
| 1. Cognitive-behavioural therapy has shown success in reducing symptoms of conduct disorder | [32–34] |
| 1. There is an association between conduct disorder and lower academic achievement | [18,29,35] |
| 1. Interventions only target the individual with the diagnosis of conduct disorder | [36–38] |
| 1. It is common for children to have both conduct disorder and attention deficit hyperactivity disorder | [39,40] |
| 1. Children with conduct disorder are less likely to drop out of an intervention if their mother is younger | [41,42] |
| 1. The diagnostic criteria for conduct disorder includes the factor “has forced somebody into sexual activity” | [12] |
| 1. The harsher the discipline a child experiences, the less likely they are to display conduct problems such as aggression and antisocial behaviour | [19,43,44] |
| 1. Interventions for conduct disorder are only carried out in clinical settings | [45,46] |
| 1. In each individual, conduct disorder is caused by either environmental risk factors or genetic risk factors, but not both | [23,47] |
| 1. All aggression shown in children with conduct disorder results from impulsivity | [48,49] |
| 1. Conduct disorder is a precursor into antisocial personality disorder in adulthood | [17] |
| 1. Simply meeting the diagnostic criteria (DSM-5) for conduct disorder makes a child eligible for placement in special education | [10,50] |
| 1. Being a victim of childhood physical abuse lowers an individual’s risk of having conduct disorder | [51,52] |
| 1. Routine pharmacological treatments are recommended for the long-term treatment of conduct disorder | [10] |
| 1. All children with conduct disorder display lack of empathy | [12,53] |
| 1. Sex is not considered a risk factor for conduct disorder because, in school age children, the prevalence is equivalent in males and females | [54,55] |
| 1. Brain impairments do not cause externalising behaviours in conduct disorder | [56,57] |
| 1. The likelihood of engaging in behaviours, such as theft, is different in child onset and adolescent onset conduct disorder | [58,59] |

Item references

1. Loy JH, Merry SN, Hetrick SE, Stasiak K. Atypical antipsychotics for disruptive behaviour disorders in children and youths. Cochrane Database Syst Rev. 2017 Aug 9;2017(8). Available from: http://doi.wiley.com/10.1002/14651858.CD008559.pub3

2. Demirkaya SK, Aksu H, Özgür BG. A Retrospective Study of Long Acting Risperidone Use to Support Treatment Adherence in Youth with Conduct Disorder. Clin Psychopharmacol Neurosci. 2017 Nov 30;15(4):328–36. Available from: http://www.cpn.or.kr/journal/view.html?doi=10.9758/cpn.2017.15.4.328

3. Frick PJ, Ray J V., Thornton LC, Kahn RE. Can callous-unemotional traits enhance the understanding, diagnosis, and treatment of serious conduct problems in children and adolescents? A comprehensive review. Psychol Bull. 2014 Jan;140(1):1–57. Available from: http://doi.apa.org/getdoi.cfm?doi=10.1037/a0033076

4. Hawes DJ, Dadds MR. The Treatment of Conduct Problems in Children With Callous-Unemotional Traits. J Consult Clin Psychol. 2005 Aug;73(4):737–41. Available from: http://doi.apa.org/getdoi.cfm?doi=10.1037/0022-006X.73.4.737

5. Hawes DJ, Price MJ, Dadds MR. Callous-Unemotional Traits and the Treatment of Conduct Problems in Childhood and Adolescence: A Comprehensive Review. Clin Child Fam Psychol Rev. 2014 Sep 19;17(3):248–67. Available from: http://link.springer.com/10.1007/s10567-014-0167-1

6. Gilmour J, Hill B, Place M, Skuse DH. Social communication deficits in conduct disorder: a clinical and community survey. J Child Psychol Psychiatry. 2004 Jul;45(5):967–78. Available from: https://onlinelibrary.wiley.com/doi/10.1111/j.1469-7610.2004.t01-1-00289.x

7. Helland WA, Posserud MB, Helland T, Heimann M, Lundervold AJ. Language Impairments in Children With ADHD and in Children With Reading Disorder. J Atten Disord. 2016 Jul 16;20(7):581–9. Available from: http://journals.sagepub.com/doi/10.1177/1087054712461530

8. Wakschlag LS. Maternal Smoking During Pregnancy and the Risk of Conduct Disorder in Boys. Arch Gen Psychiatry. 1997 Jul 1;54(7):670. Available from: http://archpsyc.jamanetwork.com/article.aspx?doi=10.1001/archpsyc.1997.01830190098010

9. Gaysina D, Fergusson DM, Leve LD, Horwood J, Reiss D, Shaw DS, et al. Maternal Smoking During Pregnancy and Offspring Conduct Problems. JAMA Psychiatry. 2013 Sep 1;70(9):956. Available from: http://archpsyc.jamanetwork.com/article.aspx?doi=10.1001/jamapsychiatry.2013.127

10. National Institute for Health and Clinical Excellence. Antisocial behaviour and conduct disorders in children and young people: Recognition, intervention and management. Nice Clinical Guideline. 2017.

11. Fairchild G, Hawes DJ, Frick PJ, Copeland WE, Odgers CL, Franke B, et al. Conduct disorder. Nat Rev Dis Prim. 2019 Dec 27;5(1):43. Available from: http://www.nature.com/articles/s41572-019-0095-y

12. American Psychiatric Association. American Psychiatric Association: Diagnostic and Statistical Manual of Mental Disorders Fifth Edition. Arlington. 2013.

13. Nock MK, Kazdin AE, Hiripi E, Kessler RC. Prevalence, subtypes, and correlates of DSM-IV conduct disorder in the National Comorbidity Survey Replication. Psychol Med. 2006 May 26;36(05):699. Available from: http://www.journals.cambridge.org/abstract_S0033291706007082

14. Petrosino A, Turpin‐Petrosino C, Hollis‐Peel ME, Lavenberg JG. Scared Straight and Other Juvenile Awareness Programs for Preventing Juvenile Delinquency: A Systematic Review. Campbell Syst Rev. 2013 Jan 2;9(1):1–55. Available from: https://onlinelibrary.wiley.com/doi/abs/10.4073/csr.2013.5

15. Tyler J, Darville R, Stalnaker K. Juvenile boot camps: a descriptive analysis of program diversity and effectiveness. Soc Sci J. 2001 Sep 1;38(3):445–60. Available from: https://www.tandfonline.com/doi/full/10.1016/S0362-3319%2801%2900130-6

16. Pringsheim T, Hirsch L, Gardner D, Gorman DA. The Pharmacological Management of Oppositional Behaviour, Conduct Problems, and Aggression in Children and Adolescents with Attention-Deficit Hyperactivity Disorder, Oppositional Defiant Disorder, and Conduct Disorder: A Systematic Review and Meta-Analysi. Can J Psychiatry. 2015 Feb;60(2):42–51. Available from: http://journals.sagepub.com/doi/10.1177/070674371506000202

17. Blair RJR, Leibenluft E, Pine DS. Conduct Disorder and Callous–Unemotional Traits in Youth. N Engl J Med. 2014 Dec 4;371(23):2207–16. Available from: http://www.nejm.org/doi/10.1056/NEJMra1315612

18. Miech RA, Caspi A, Moffitt TE, Wright BRE, Silva PA. Low Socioeconomic Status and Mental Disorders: A Longitudinal Study of Selection and Causation during Young Adulthood. Am J Sociol. 1999 Jan;104(4):1096–131. Available from: https://www.journals.uchicago.edu/doi/10.1086/210137

19. Moore AA, Silberg JL, Roberson-Nay R, Mezuk B. Life course persistent and adolescence limited conduct disorder in a nationally representative US sample: prevalence, predictors, and outcomes. Soc Psychiatry Psychiatr Epidemiol. 2017 Apr 8;52(4):435–43. Available from: http://link.springer.com/10.1007/s00127-017-1337-5

20. Piotrowska PJ, Stride CB, Croft SE, Rowe R. Socioeconomic status and antisocial behaviour among children and adolescents: A systematic review and meta-analysis. Clin Psychol Rev. 2015 Feb;35:47–55. Available from: https://linkinghub.elsevier.com/retrieve/pii/S0272735814001652

21. Salvatore JE, Dick DM. Genetic influences on conduct disorder. Neurosci Biobehav Rev. 2018 Aug;91:91–101. Available from: https://linkinghub.elsevier.com/retrieve/pii/S0149763416300835

22. Wesseldijk LW, Bartels M, Vink JM, van Beijsterveldt CEM, Ligthart L, Boomsma DI, et al. Genetic and environmental influences on conduct and antisocial personality problems in childhood, adolescence, and adulthood. Eur Child Adolesc Psychiatry. 2018 Sep 21;27(9):1123–32. Available from: http://link.springer.com/10.1007/s00787-017-1014-y

23. Jaffee SR, Caspi A, Moffitt TE, Dodge KA, Rutter M, Taylor A, et al. Nature × nurture: Genetic vulnerabilities interact with physical maltreatment to promote conduct problems. Dev Psychopathol. 2005 Mar 7;17(01). Available from: http://www.journals.cambridge.org/abstract_S0954579405050042

24. Chronis AM, Lahey BB, Pelham WE, Williams SH, Baumann BL, Kipp H, et al. Maternal depression and early positive parenting predict future conduct problems in young children with attention-deficit/hyperactivity disorder. Dev Psychol. 2007 Jan;43(1):70–82. Available from: http://doi.apa.org/getdoi.cfm?doi=10.1037/0012-1649.43.1.70

25. Kim-Cohen J, Moffitt TE, Taylor A, Pawlby SJ, Caspi A. Maternal Depression and Children’s Antisocial Behavior. Arch Gen Psychiatry. 2005 Feb 1;62(2):173. Available from: http://archpsyc.jamanetwork.com/article.aspx?doi=10.1001/archpsyc.62.2.173

26. Leschied AW, Chiodo D, Whitehead PC, Hurley D. The relationship between maternal depression and child outcomes in a child welfare sample: implications for treatment and policy. Child <html_ent glyph="@amp;" ascii="&amp;"/> Fam Soc Work. 2005 Nov;10(4):281–91. Available from: https://onlinelibrary.wiley.com/doi/10.1111/j.1365-2206.2005.00365.x

27. Canino G, Polanczyk G, Bauermeister JJ, Rohde LA, Frick PJ. Does the prevalence of CD and ODD vary across cultures? Soc Psychiatry Psychiatr Epidemiol. 2010 Jul 9;45(7):695–704. Available from: http://link.springer.com/10.1007/s00127-010-0242-y

28. Galler JR, Bryce CP, Waber DP, Hock RS, Harrison R, Eaglesfield GD, et al. Infant malnutrition predicts conduct problems in adolescents. Nutr Neurosci. 2012 Jul 19;15(4):186–92. Available from: http://www.tandfonline.com/doi/full/10.1179/1476830512Y.0000000012

29. Erskine HE, Norman RE, Ferrari AJ, Chan GCK, Copeland WE, Whiteford HA, et al. Long-Term Outcomes of Attention-Deficit/Hyperactivity Disorder and Conduct Disorder: A Systematic Review and Meta-Analysis. J Am Acad Child Adolesc Psychiatry. 2016 Oct;55(10):841–50.

30. Farrington DP, Coid J, Harnett L, Jolliffe D, Soteriou N, Turner R, et al. Criminal Careers Up to Age 50 and Life Success Up to Age 48: New Findings from the Cambridge Study in Delinquent Development. Home Off Res Study. 2006 Jan 1;299.

31. Farrington DP. The Development of Offending and Antisocial Behaviour from Childhood: Key Findings from the Cambridge Study in Delinquent Development. J Child Psychol Psychiatry. 1995 Sep;36(6):929–64. Available from: https://onlinelibrary.wiley.com/doi/10.1111/j.1469-7610.1995.tb01342.x

32. Sukhodolsky DG, Kassinove H, Gorman BS. Cognitive-behavioral therapy for anger in children and adolescents: a meta-analysis. Aggress Violent Behav. 2004 May;9(3):247–69. Available from: https://linkinghub.elsevier.com/retrieve/pii/S1359178903000727

33. Olanike A. Cognitive Behaviour Therapy in the Management of Conduct Disorder Among Adolescents. In: Mental Disorders - Theoretical and Empirical Perspectives. InTech; 2013. Available from: http://www.intechopen.com/books/mental-disorders-theoretical-and-empirical-perspectives/cognitive-behaviour-therapy-in-the-management-of-conduct-disorder-among-adolescents

34. McCart MR, Sheidow AJ. Evidence-Based Psychosocial Treatments for Adolescents With Disruptive Behavior. J Clin Child Adolesc Psychol. 2016 Sep 2;45(5):529–63. Available from: https://www.tandfonline.com/doi/full/10.1080/15374416.2016.1146990

35. Colman I, Murray J, Abbott RA, Maughan B, Kuh D, Croudace TJ, et al. Outcomes of conduct problems in adolescence: 40 year follow-up of national cohort. BMJ. 2009 Jan 8;338(jan08 2):a2981–a2981. Available from: https://www.bmj.com/lookup/doi/10.1136/bmj.a2981

36. Comer JS, Chow C, Chan PT, Cooper-Vince C, Wilson LAS. Psychosocial Treatment Efficacy for Disruptive Behavior Problems in Very Young Children: A Meta-Analytic Examination. J Am Acad Child Adolesc Psychiatry. 2013 Jan;52(1):26–36. Available from: https://linkinghub.elsevier.com/retrieve/pii/S089085671200768X

37. Woolfenden SR. Family and parenting interventions for conduct disorder and delinquency: a meta-analysis of randomised controlled trials. Arch Dis Child. 2002 Apr 1;86(4):251–6. Available from: https://adc.bmj.com/lookup/doi/10.1136/adc.86.4.251

38. van der Stouwe T, Asscher JJ, Stams GJJM, Deković M, van der Laan PH. The effectiveness of Multisystemic Therapy (MST): A meta-analysis. Clin Psychol Rev. 2014 Aug;34(6):468–81. Available from: https://linkinghub.elsevier.com/retrieve/pii/S0272735814000981

39. Gnanavel S, Sharma P, Kaushal P, Hussain S. Attention deficit hyperactivity disorder and comorbidity: A review of literature. World J Clin Cases. 2019 Sep 6;7(17):2420–6. Available from: https://www.wjgnet.com/2307-8960/full/v7/i17/2420.htm

40. Connor DF, Ford JD, Albert DB, Doerfler LA. Conduct Disorder Subtype and Comorbidity. Ann Clin Psychiatry. 2007 Jul;19(3):161–8. Available from: http://www.portico.org/Portico/article?article=pf1m9kdb8p

41. Kazdin AE, Mazurick JL, Bass D. Risk for Attrition in Treatment of Antisocial Children and Families. J Clin Child Psychol. 1993 Mar 7;22(1):2–16. Available from: http://www.tandfonline.com/doi/abs/10.1207/s15374424jccp2201_1

42. Luk ESL, Staiger PK, Mathai J, Wong L, Birleson P, Adler R. Children with persistent conduct problems who dropout of treatment. Eur Child Adolesc Psychiatry. 2001 Mar 21;10(1):28–36. Available from: http://link.springer.com/10.1007/s007870170044

43. Bender HL, Allen JP, McElhaney KB, Antonishak J, Moore CM, Kelly HO, et al. Use of harsh physical discipline and developmental outcomes in adolescence. Dev Psychopathol. 2007 Jan 22;19(01). Available from: http://www.journals.cambridge.org/abstract_S0954579407070125

44. Jaffee SR, Strait LB, Odgers CL. From correlates to causes: Can quasi-experimental studies and statistical innovations bring us closer to identifying the causes of antisocial behavior? Psychol Bull. 2012;138(2):272–95. Available from: http://doi.apa.org/getdoi.cfm?doi=10.1037/a0026020

45. Scott S. An update on interventions for conduct disorder. Adv Psychiatr Treat. 2008 Jan 2;14(1):61–70. Available from: https://www.cambridge.org/core/product/identifier/S1355514600004417/type/journal_article

46. Pilling S, Gould N, Whittington C, Taylor C, Scott S. Recognition, intervention, and management of antisocial behaviour and conduct disorders in children and young people: summary of NICE-SCIE guidance. BMJ. 2013 Mar 27;346(mar27 1):f1298–f1298. Available from: https://www.bmj.com/lookup/doi/10.1136/bmj.f1298

47. Dodge KA. Mechanisms of Gene-Environment Interaction Effects in the Development of Conduct Disorder. Perspect Psychol Sci. 2009 Jul;4(4):408–14. Available from: http://journals.sagepub.com/doi/10.1111/j.1745-6924.2009.01147.x

48. Mathias CW, Stanford MS, Marsh DM, Frick PJ, Moeller FG, Swann AC, et al. Characterizing aggressive behavior with the Impulsive/Premeditated Aggression Scale among adolescents with conduct disorder. Psychiatry Res. 2007 Jun;151(3):231–42. Available from: https://linkinghub.elsevier.com/retrieve/pii/S0165178106003623

49. Dougherty DM, Dew RE, Mathias CW, Marsh DM, Addicott MA, Barratt ES. Impulsive and premeditated subtypes of aggression in conduct disorder: differences in time estimation. Aggress Behav. 2007 Nov;33(6):574–82. Available from: https://onlinelibrary.wiley.com/doi/10.1002/ab.20219

50. Hughes TL, Crothers LM, Jimerson SR. Identifying, Assessing, and Treating Conduct Disorder at School. Springer; 2008.

51. Kaplan SJ, Pelcovitz D, Salzinger S, Weiner M, Mandel FS, Lesser ML, et al. Adolescent Physical Abuse: Risk for Adolescent Psychiatric Disorders. Am J Psychiatry. 1998 Jul;155(7):954–9. Available from: http://psychiatryonline.org/doi/abs/10.1176/ajp.155.7.954

52. Norman RE, Byambaa M, De R, Butchart A, Scott J, Vos T. The Long-Term Health Consequences of Child Physical Abuse, Emotional Abuse, and Neglect: A Systematic Review and Meta-Analysis. Tomlinson M, editor. PLoS Med. 2012 Nov 27;9(11):e1001349. Available from: https://dx.plos.org/10.1371/journal.pmed.1001349

53. Milone A, Cerniglia L, Cristofani C, Inguaggiato E, Levantini V, Masi G, et al. Empathy in Youths with Conduct Disorder and Callous-Unemotional Traits. Neural Plast. 2019 Apr 11;2019:1–8. Available from: https://www.hindawi.com/journals/np/2019/9638973/

54. Erskine HE, Ferrari AJ, Nelson P, Polanczyk G V., Flaxman AD, Vos T, et al. Research Review: Epidemiological modelling of attention-deficit/hyperactivity disorder and conduct disorder for the Global Burden of Disease Study 2010. J Child Psychol Psychiatry. 2013 Dec;54(12):1263–74. Available from: http://doi.wiley.com/10.1111/jcpp.12144

55. NHS Digital. Mental Health of Children and Young People in England, 2017. 2018. Available from: https://files.digital.nhs.uk/A6/EA7D58/MHCYP 2017 Summary.pdf

56. Zhou J, Yao N, Fairchild G, Zhang Y, Wang X. Altered Hemodynamic Activity in Conduct Disorder: A Resting-State fMRI Investigation. Zhan W, editor. PLoS One. 2015 Mar 27;10(3):e0122750. Available from: https://dx.plos.org/10.1371/journal.pone.0122750

57. Alegria AA, Radua J, Rubia K. Meta-Analysis of fMRI Studies of Disruptive Behavior Disorders. Am J Psychiatry. 2016 Nov;173(11):1119–30. Available from: http://ajp.psychiatryonline.org/doi/10.1176/appi.ajp.2016.15081089

58. Moffitt TE, Arseneault L, Jaffee SR, Kim-Cohen J, Koenen KC, Odgers CL, et al. Research Review: DSM-V conduct disorder: research needs for an evidence base. J Child Psychol Psychiatry. 2008 Jan;49(1):3–33. Available from: https://onlinelibrary.wiley.com/doi/10.1111/j.1469-7610.2007.01823.x

59. Lahey BB, Goodman SH, Waldman ID, Bird H, Canino G, Jensen P, et al. Relation of Age of Onset to the type and Severity of Child and Adolescent Conduct Problems. J Abnorm Child Psychol. 1999;27:247–60.
