# Supplementary material for: Knowledge and attitudes about conduct disorder of professionals working with young people: The influence of occupation and direct and indirect experience
Source: PLoS One. 2023 Sep 28;18(9):e0292271. doi: 10.1371/journal.pone.0292271 (PMC10538712; doi:10.1371/journal.pone.0292271)
Supplement: Attachment — Submitted filename: Letter to Reviewers.docx [file pone.0292271.s002.docx]

18^th^ August 2023

Dear Professor Lund and Reviewer 1,

Thank you for your much-appreciated comments on the manuscript. Your feedback has been addressed, as below:

**Editors feedback**

**Please retain information about the prize draw and confidentiality of emails in the revision (disregard this one comment from the reviewer).**

This information has been retained.

**In addition to including the eligibility criteria (as requested by Reviewer 1), please also justify how the 10-17 age range for clients/patients was chosen.**

This has been addressed in the paper (page 8, line 187), as highlighted in red below.

*Participants were required to provide informed consent and to be working with young people aged 10-17 in the UK. This age range was chosen to achieve a balance between maximising sample size and consistency across the clinical population in question, i.e. to include pre-adolescent and adolescent youth but not very young children.*

**Please include information on the percentage and number of participants with missing data, how missing data were handled, and why the method of addressing missing data was chosen.**

This has been addressed in the manuscript (page 11, line 288).

*Some data was missing from attitude component questions, so participants’ responses were only included for the components they answered to ensure maximum data inclusion. Calculations were carried out on the following number of remaining participants: stereotypic beliefs (n=50, missing 8 [13.79%]), symbolic beliefs (n=47, missing 11 [18.97%]), affect (n=48, missing 10 [17.24%]), and past behaviour (n=36, missing 22 [37.93%]).*

**Please clarify if the knowledge measure was developed for this study or is pre-existing. Please explain how the measure was developed and checked for accuracy and include the measure as an appendix if newly developed.**

Materials section has been updated (page 10, line 244), as highlighted in red below. The measure has also been added to the Appendices.

*The knowledge measure was adapted from Anderson et al. [26]. New questions were developed, drawing from literature on relevant items, using the original format of 33 questions (see Appendix for the tool, its format, and references). There were three 11-item subscales exploring knowledge of (i) causes, (ii) treatments, and (iii) characteristics. The order of questions for each subscale and true answers remained the same as the original knowledge measure by Anderson et al. [26].*

**Reviewer 1 feedback**

**The study is incorrectly described as a between subjects design, but the statistical analysis used is appropriate for a cross-sectional survey design.**

The use of ‘between-subjects’ was an oversight, which has been corrected to cross-sectional (page 8, line 204).

**Information about the email addresses and prize draw isn't necessary for this paper.**

This has been retained as suggested by editor, see first point.

**Eligibility criteria for participants should be made more explicit.**

This has been addressed in the paper (page 9, line 219).

*Participants were required to provide informed consent and to be working with young people aged 10-17 in the UK. This age range was chosen to achieve a balance between maximising sample size and consistency across the clinical population in question, i.e. to include pre-adolescent and adolescent youth but not very young children.*

**How was the study size arrived at? If a sample size calculation was performed, please state.**

The power-analysis calculation has been added (page 7, line 185), as below.

*To obtain 80% power, allowing for a detection of a large effect size of 0.8, with a significance threshold of p<0.05, at least 24 participants were required for one-way Analysis of Variance (ANOVA) tests and 52 participants for two-way independent sample t-tests, calculated by G*Power.*

**Please provide more detail about recruitment methods - clarify if recruiting through specific groups and networks as this can be relevant to generalisability of findings.**

More information on recruitment has been added (page 8, line 193), as highlighted in red below.

*Advertisements for the study were posted and promoted on the researchers’ main and professional accounts on social media (Facebook, LinkedIn, and Twitter), as well as various research and dissertation groups on Facebook. Advertisement posts were also shared by various other members of the public to reach a wider audience.*

**Please provide more information about the survey. For example, what demographic information was collected? What information was collected about experience? Detail any pilot testing that was done.**

More information now in Materials (page 9, line 227), as highlighted in red below.

*The following demographic information was collected: gender, age, where they work with young people, whether the setting is forensic or non-forensic, their main job role, whether their work is full-time/part-time/bank, and years of employment in this role. Education and training were accounted for through asking whether they had completed any university units or training on CD. Experience with CD was measured through asking whether they have worked with a child believed to have CD.*

**Please provide more information about how open-ended responses were analysed, that is, how did you group the responses? Only the scoring information is provided.**

This has also been updated in Materials (page 11, line 269), as highlighted in red below.

*Participants wrote up to 12 words or phrases answering each component statement, relevant to CD. Participants transformed their own responses into quantitative data themselves by rating each word or phrase on a 5-point scale ranging from ‘very negative’ (-2) to ‘very positive’ (+2). Each participant’s four individual scores for each of the four components were formed by calculating a mean score of the ratings given to their responses to each statement.*

**Provide information on representativeness of the sample - this is mentioned in discussion but should be stated clearly in your results.**

Now addressed in Results (page 12, line 316), as highlighted in red below.

*It is important to highlight that the sample size is relatively small. It is also predominantly female, however this may be representative of the number of females working with CD in the general population [42].*

**In the limitations it says that the study was powered to detect large to moderate effects - please correct this language as no power analysis is provided in your methods section, and power analysis may be incorrect based on the study design stated in the methods.**

The power-analysis calculation has been checked and added, as addressed above (page 7, line 185).

**Sample size is a major limitation of this study - can you provide details for why the sample was so small? the population being drawn from is over 1 million (teachers in UK alone are about 500K), so having only 58 participants in this study bears some introspection. Detail about recruitment methods will also help understand the generalisability - if recruiting through networks with more CD experience/interest this is very relevant.**

This has been addressed in the discussion (page 22, line 548), as highlighted below.

*Despite this study being promoted and widely circulated online, uptake and completion was limited to a relatively small sample of 58. There are several possible reasons for this, two of which warrent mention. Firstly, it may be that many staff working with CD are unaware or unclear about of the concept of CD, because it is rarely diagnosed [13], and so the advert would not have captured their attention. This highlights the importance of psychoeducation about CD for staff within educational and healthcare settings for young people. Secondly, CD is a contentious issue*. *Some argue that CD does not exist and the diagnosis is harmful, whereas others believe a diagnosis can help highlight treatment needs [64].* *Even within groups of staff who are aware of the CD concept, some individuals who work with youths with CD may be reluctant to validate the concept by engaging in research using this terminology. Frank and open discussions about the quality of evidence for CD and the potential ethical and practical implications of use of this diagnosis are important for researchers in this field to embrace.*

**Please change biological sex to gender or omit the word 'biological'.**

The word ‘biological’ has been omitted (page 23, line 572).

**Data accessibility**

**We note that you have indicated that data from this study are available upon request. PLOS only allows data to be available upon request if there are legal or ethical restrictions on sharing data publicly.**

Data is available at <http://doi.org/10.17639/nott.7331>. Email addresses and demographic data have been omitted, as stated in the data sharing section of the consent form.

While reviewing the text, we have made some further very minor amendments for style/clarity, which we hope are acceptable, as below.

Formatting, including citation format and heading levels, have been corrected throughout.

Adjustments have been made to the authors’ information (page 1, line 6), highlighted in red below.

*^1^ Centre for Forensic and Family Psychology, School of Medicine, University of Nottingham, Nottingham, United Kingdom*

*^2^ Clayfields House, Nottinghamshire County Council, Nottingham, United Kingdom*

*^3^ Centre for Human Brain Health, School of Psychology, University of Birmingham, Birmingham, United Kingdom*

*^4^ Academic Unit of Mental Health and Clinical Neurosciences, School of Medicine, University of Nottingham, Nottingham, United Kingdom*

*^5^ Nottinghamshire Healthcare NHS Trust, United Kingdom*

Clarity added to statement in introduction (page 5, line 111), as below.

*Other data suggest that psychiatrists have varied knowledge of the aetiologies of CD [25].*

Sentence format, grammar, and punctuation have been adjusted in the following areas.

- Page 5, line 126:

*Some studies suggest that teachers view children with CD as disruptive and have concerns about the safety of other students during lessons [22–24], indicating negative beliefs.*

- Page 6, line 134 (the word formal has been removed from “a lack of formal statistical appraisal”):

*Inferences from this work are limited, however, due to a lack of statistical appraisal of findings.*

- Page 6, line 144:

*Another study [26] adapted open-ended response measures from Haddock & Zanna [33] and demonstrated that experience elicits differences in knowledge and attitude components.*

- Page 6, line 152 (colon removed from after “such as”):

*Research demonstrates that direct experience of working in general mental health settings reduces negative attitudes towards patients because unrealistic preconceptions are challenged, such as the fear that psychiatric patients pose a threat and people with diagnoses are out of control [36].*

- Page 6, line 155:

*Although evidence relating to CD is limited, qualitative work shows that direct experience of teaching children with disruptive behaviour disorders, including CD, contributes to greater confidence in teachers’ knowledge about the disorders and management techniques for these [22,23].*

- Page 7, line 171:

*Drawing on a limited previous literature, we hypothesised that (i)* *Psychology Staff would have significantly greater knowledge, favourable global attitudes, and favourable attitude components (tripartite model) than other staff groups; (ii) individuals with direct experience with young people with CD would have significantly greater knowledge, favourable global attitudes, and favourable attitude components than individuals without direct experience, across the entire sample (our design was not powered to perform within-group analyses); and (iii) individuals with indirect experience of CD (training/education) would have greater knowledge, favourable global attitudes, and favourable attitude components than individuals without indirect experience, across occupational groups.*

- Page 8, line 200:

*One doctor, who did not fit into any of these four occupation groups, was excluded, resulting in an overall sample of 58 participants.*

- Page 10, line 246:

*There were three 11-item subscales exploring knowledge of (i) causes, (ii) treatments, and (iii) characteristics.*

- Page 12, line 315:

*It is important to highlight that the sample size is relatively small.*

- Page 18, line 414:

*Thirdly, those with indirect experience (training or education) about CD had significantly greater objective knowledge and global attitudes than those without, though there were no significant differences in any attitude subcomponents.*

- Page 19, line 460:

*supporting the view that occupation-based experiences may impact global attitudes.*

- Page 19, line 464:

*This is supported by previous research demonstrating that teachers report many concerns when teaching children with CD [22,23],*

- Page 20, line 490:

*Therefore, controlling for types of training methods would provide greater insight into the impact it has on knowledge.*

- Page 21, line 497 (the word thus has also been removed within “may thus explain”):

*This may result from empathic responses to individuals with CD, where professionals may explain behaviours as a result of a diagnosis of CD [54]. Furthermore, those who seek out training in CD may have pre-existing favourable attitudes to CD. Supporting this, previous findings suggest a relationship between having more empathy and a greater understanding of mental health issues [54].*

- Page 21, line 506:

*Therefore, their experience may not be sufficient enough to change beliefs.*

- Page 22, line 528:

*On the other hand, it may cause professionals to be guarded about CD, and develop a therapeutic hopelessness about outcomes for the disorder, seeing affected individuals as beyond the influence of usual psychosocial interventions.*

Finally, the following paragraph was deleted due to it being addressed earlier in the section when discussing sample size:

*A final potential limitation is that the demographic questionnaire asked whether staff have worked with individuals ‘believed to have CD’ because CD is rarely diagnosed [13], potentially causing an incorrect estimation of individuals with direct experience. Therefore, future research may benefit from focusing on attitudes towards youths with a confirmed clinical diagnosis.*

Thank you again for your help. I look forward to hearing back from you.

Kind regards,

Chloe Pinchess

*Corresponding author*

[*chloepinchess@icloud.com*](mailto:chloepinchess@icloud.com)
